# Development and psychometric properties of a brief measure of subjective decision quality for breast cancer treatment

**DOI:** 10.1186/s12911-014-0110-x

**Published:** 2014-12-05

**Authors:** Ken Resnicow, Paul Abrahamse, Rachel S Tocco, Sarah Hawley, Jennifer Griggs, Nancy Janz, Angela Fagerlin, Adrienne Wilson, Kevin C Ward, Sheryl GA Gabram, Steven Katz

**Affiliations:** University of Michigan, School of Public Health, 109 Observatory Street, Ann Arbor, MI 48109-2029 USA; University of Michigan, School of Medicine, 2800 Plymouth Road, Ann Arbor, MI 48109 USA; Veterans Administration, Ann Arbor Center for Clinical Management Research, 2800 Plymouth Road, Building 16, Rm. 421 W, Ann Arbor, MI 48109-2800 USA; Emory University, Rollins School of Public Health, 1518 Clifton Road, NE, Atlanta, GA 30322 USA; Department of Surgery, Emory University School of Medicine, Winship Cancer Institute of Emory University, 69 Jesse Hill Jr. Dr. SE, Room 303, Atlanta, GA 30303 USA

**Keywords:** Breast cancer, Oncology, Decision making, Decision quality, Decision satisfaction, Scale development, Psychometric testing

## Abstract

**Background:**

Breast cancer patients face several preference-sensitive treatment decisions. Feelings such as regret or having had inadequate information about these decisions can significantly alter patient perceptions of recovery and recurrence. Numerous objective measures of decision quality (e.g., knowledge assessments, values concordance measures) have been developed; there are far fewer measures of subjective decision quality and little consensus regarding how the construct should be assessed. The current study explores the psychometric properties of a new subjective quality decision measure for breast cancer treatment that could be used for other preference sensitive decisions*.*

**Methods:**

320 women aged 20–79 diagnosed with AJCC stage 0 – III breast cancer were surveyed at two cancer specialty centers. Decision quality was assessed with single items representing six dimensions: regret, satisfaction, and fit as well as perceived adequacy of information, time, and involvement. Women rated decision quality for their overall treatment experience and surgery, chemotherapy, and radiation decisions separately. Principle components was used to explore factor structure. After scales were formed, internal consistency was computed using Cronbach’s alpha. The association of each of the four final scales with patient characteristics scores was examined by Pearson correlation.

**Results:**

For overall breast cancer treatment as well as surgery, chemotherapy, and radiation decisions, the six items yielded a single factor solution. Factor loadings of the six decision items were all above .45 across the overall and treatment-specific scales, with the exception of “Right for You” for chemotherapy and radiation. Internal consistency was 0.77, 0.85, 0.82, and 0.78 for the overall, surgery, chemotherapy, and radiation decision quality scales, respectively.

**Conclusions:**

Our measure of subjective appraisal of breast cancer treatment decisions includes 5 related elements; regret and satisfaction as well as perceived adequacy of information, time, and involvement. Future research is needed to establish norms for the measure as is further psychometric testing, particularly to examine how it is associated with outcomes such as quality of life, psychological coping and objective decision quality.

## Background

Patient appraisal of breast cancer treatment decisions is an important component of psychological coping and quality of life [1-4]. Breast cancer (BC) patients face numerous complex and consequential decisions regarding diagnostic and genetic testing, local and systemic treatment, and the possibility of reconstructive surgery. The process of making BC treatment decisions can engender anxiety and fear in the immediate period following diagnosis. Over time, feelings such as regret or having had inadequate information, time or input about these important decisions can significantly alter perceptions of recovery and recurrence [5-7]. Decision satisfaction is particularly important in breast cancer because many treatment decisions, such as the choice to undergo mastectomy versus breast conserving surgery, are preference sensitive. That is, they are (or at least should be) driven more by patient preferences and values than any clear medical advantage [8-10]. There is a need therefore, for valid measures to help researchers and clinicians assess how patients perceive the quality of their BC treatment decisions [11].

Approaches to measuring decision quality for BC can be dichotomized into “objective” and “subjective” methods [8,9,12]. Objective measures of decision-making generally focus on quantifying the decision process. This includes assessment of factors such as patient knowledge, e.g., awareness that breast conserving surgery and mastectomy generally yield similar survival rates or the potential need for chemotherapy following surgery The assumption is that decisions made by informed patients are inherently higher quality than those based on inaccurate or insufficient information [2,8,13,14]. Another component of objective assessment of treatment decisions entails rating the concordance/dissonance between the patient’s preferred decision making style, e.g., patient driven versus provider driven, and how the treatment decisions were actually made [1-4,9,13]. Concordance also includes how well the selected treatments or tests match patients’ values or preferences, e.g., a patient who desires to keep her natural breast over other treatment factors should receive lumpectomy [8]. Here again, the assumption is that higher concordance between patient preferences and either the decision process utilized or the treatment and tests received represents a higher quality decision [13,14]. And finally, while the ability to quantify the nature of the decision (e.g., high or low) is a strength of objective measures, obtaining a true objective assessment of decision making is often challenging (e.g., determining if the decision was values concordant) and can bleed over into more qualitative assessment.

Subjective assessment of decision quality, on the other hand, focuses on patients’ appraisal of the decision making process. Subjective decisional quality has been operationalized using several dimensions, most commonly satisfaction and regret [13,15-18]. Although there is no “correct” response for this type of measure, as there is in objective measures, the assumption is that higher subjective satisfaction and lower regret represent higher quality decisions. Aspects of “objective” quality decisions can be incorporated into subjective measures by querying patient perceptions of the adequacy of their knowledge, their level of involvement, or the amount of time they had to make their decisions [9,15-17,19-23]. Rather than focusing on what patients know, subjective knowledge assessment addresses their perceived adequacy of information, which may be high even absent key information or low even when key information is known. The same holds true for subjective rating of the degree of involvement and the amount of time they had to make their decisions. Objective and subjective assessments appear to tap different dimensions of decision quality, as there is often only a weak association between subjective ratings of knowledge and actual knowledge [24,25]. Little has been reported on the association between objective and subjective concordance.

Although the distinction between objective and subjective elements of breast cancer (or more broadly, medical) decision making has not been previously proposed, this dichotomy mirrors the distinction between the deliberation process and the determination of decisions proposed by Elwyn and Miron-Shatz [26]. Specifically the authors note (ibid page 143), “we need to evaluate both the decision making (the perceived or observed process) and the determination (whether or not the decision itself is considered “good”)”. Our proposed measure similarly distinguishes between the objective processes used in making the decision and the subsequent subjective evaluation of that decision, with a focus on the latter. Similarly, Sepucha et al., delineate two primary dimension for evaluating decision aids, **1)** the quality of the decision-making process and, **2)** the quality of the choice [27].

Numerous objective knowledge and concordance measures, particularly for the purpose of evaluating patient decision aids [8,27,28]*,* have been reported [9,12,16,19] including the Decisional Conflict Scale [29,30] and some consensus regarding the content of these measures has emerged [27]. There are, however, fewer measures of subjective decision quality and even less consensus regarding how the construct should be assessed. We could find only one measure that exclusively tapped subjective decisional quality (SDQ); the Satisfaction With Decision (SWD) scale by Holmes-Rovner et al. [31]. This measure however, only assesses positive aspects of decision quality (DQ), i.e., it contains no negatively framed items such as regret and does not assess satisfaction with how much involvement and time the patient perceives they had in making their decision, both of which have been proposed as key elements of a quality decision [11]. The proposed measure evaluated herein, includes these elements.

The primary aim of this study was to explore the psychometric properties of a new subjective quality decision measure for breast cancer treatment decision quality that could be applied to other preference sensitive decisions. The goal was to produce a brief yet comprehensive measure that can be used by researchers and practitioners.

Conceptually, our measure was also informed by Self-Determination Theory (SDT), which distinguishes between controlled and autonomous motivation [32-34]. SDT posits that behavior change decisions that respect and support the patient’s autonomy and competence are more likely to be perceived as higher quality than decisions that leave the patient feeling pressured or compliant (i.e., controlled motivation). Specifically, the item regarding adequate knowledge is intended to tap competence, whereas items related to involvement and time are intended to tap the degree of autonomy in the decision making process.

Based on our theoretical framework and our team’s experience [17] as well as a review of the literature and extant measures [4,7,20,21] we generated six potential dimensions of subjective decision quality: regret, satisfaction, and fit (operationalized by asking if the treatment received was “right for you”) as well as perceived adequacy of information, time, and involvement in making their breast cancer treatment decisions. Decision quality was assessed for the patient’s overall treatment decisions, as well as for surgery, chemotherapy, and radiation decisions separately.

## Methods

### Study sample and data collection

The target sample was women aged 20–79 who were diagnosed with American Joint Committee on Cancer (AJCC) stage 0 – III breast cancer within the previous 18 months. Participants were recruited from Memorial Sloan-Kettering Cancer Center (MSKCC) in New York and from Emory Midtown Hospital, the Winship Cancer Institute of Emory University, and Grady Memorial Hospital in Georgia between June and September 2013. Based on sample size calculations for both precision of prevalence estimates (there were other items being tested on the pilot survey not reported here) as well as sample adequacy for factor analysis, we set a quota sample of 250 completed surveys and over-recruited to ensure adequate statistical power.

At MSKCC, eligible breast cancer patients were approached in-clinic and asked to complete the survey. Patients who met the inclusion criteria were identified by examining the clinic schedule for the upcoming day and when feasible, all eligible patients who showed up were approached. These women were given the option to take the survey home to complete if requested. A $10 incentive was provided to respondents upon completion of their survey. Individuals who did not respond received a reminder call, if applicable, re-approached if they had a return visit to the clinic during the piloting timeframe.

At the Georgia sites, eligible breast cancer patients were identified by clinic records and mailed a survey packet which included a $10 pre-incentive. Reminder postcards and follow-up phone calls were made to non-responders and second survey packets (with no second incentive) were mailed as needed. The institutional review boards of the University of Michigan, MSKCC, and Emory University approved all study procedures and materials and all participants gave their informed consent prior to their inclusion in the study.

The response rate was 77.8% (214 of 275) in New York and 56.7% (140 of 247) in Georgia, for a combined response rate of 67.8% (354/522). We excluded from all analyses 30 patients who were more than 18 months after diagnosis (to maximize recall accuracy) and four who had completely missing data, leaving a final sample of 320. For factor and internal consistency analysis by treatment modality, which required all items to be completed, the final sample size was 286, 286, 195, and 247, for the overall treatment experience (hereafter referred to as ‘overall”), surgery, chemotherapy, and radiation scales respectively. In addition, when computing scale means, we excluded those who had two or more missing items as well as those who indicated that the procedure was not offered. There were 22, 25, 113, and 65 surveys that were excluded from the overall, surgery, chemotherapy and radiation therapy scales, respectively for these reasons. Thus, the final sample size for scale means was 298, 295, 207, and 255, for the overall, surgery, chemotherapy, and radiation scales.

The number endorsing “not offered” across the six items for overall treatment ranged from 2 to 8, for surgery, 7 to 12, for radiation, 47 to 57, and for chemotherapy, 83 to 102. The skip rate across the six items for overall treatment ranged from 2 to 8, for surgery 2 to 6, for radiation, 5 to 12, and for chemotherapy 3 to 6 respondents. Data not shown.

### Measures

#### Patient characteristics

Participants were asked about their age (dichotomized around the median of 58 years), race, ethnicity, and level of education (collapsed into high school or less, some college, or college or higher) as well as the amount of time (in months) since their breast cancer diagnosis (dichotomized as less than or equal to or greater than 12 months). We also asked yes/no questions to ascertain whether or not they had received various tests or treatments, specifically breast MRI, imaging tests (bone scan, PET scan, or CT scan), BRCA genetic testing, the 21-gene assay (Oncotype DX®), lumpectomy, mastectomy, radiation therapy, and chemotherapy.

#### Decision quality

Decision quality was assessed initially with six dimensions, each comprising one item. For each dimension we asked the respondent to rate “overall treatment for your breast cancer” as well as their decision about “which type of surgery to have”, “whether or not to have chemotherapy”, and “whether or not to have radiation therapy”. The overall item was placed before surgery, chemotherapy, and radiation items for the information, involvement, and satisfaction sections of the survey and afterwards for the “right for you”, time, and regret sections. The six dimensions were assessed as described in Table 1 below:

**Table 1:** **Decision quality instrument**

a.	How “right for you” were the following decisions?^*^
b.	How much information did you have for the following decisions?^+^
c.	How much time did you have for the following decisions?^+^
d.	How much involvement did you have in the following decisions?^+^
e.	**How much regret do you have about the following decisions?** ^**^**^
f.	How satisfied are you with the following decisions?^^^

^*^Rated on a 7-point scale anchored with: Not at all right for me (1), Neutral (4), and Completely right for me (7).

^+^Rated on a 7-point scale anchored with: Not enough (1), Just Right (4), and Too Much (7).

^^^Rated on a 7-point scale anchored with: No regret/Not at All Satisfied (1), Some regret/Somewhat Satisfied (4), and A lot of regret/Totally Satisfied (7).

Bolded item is reverse-coded.

N/A option also included for all items.

For adequacy of information, time, and involvement we recoded values in two ways. First we recoded values 5 to 7 into 3 to 1, respectively, as we considered both “not enough“ and “too much” an equally low quality decision compared to “just right”, which was given the highest value of 4. Next, we recoded these 1 to 4 values to match the seven point scaling used in the remaining three items, so that (1 = 1) (2 = 3) (3 = 5) and (4 = 7). In addition, the regret item was reverse coded so that for all items and scale scores higher values indicated higher subjective decision quality.

### Analyses

Principle components analysis was used to explore factor structure and determine the appropriate number of factors. We considered retaining factors with Eigen values >1.0. We used item loading values of .45 or higher as indication that the item should be retained in the final scale. After scales were formed we computed internal consistency using Cronbach’s alpha, and reported the alpha with each item removed. The association of each of the four final scale scores was examined by Pearson correlation. In addition to exploring item-level psychometrics we also explored the association of scale scores for decisions regarding surgery, chemotherapy, and radiation as well as the association of mean scale scores by patient characteristics using a t-test. All analyses for this paper were generated using SAS software.

## Results

As shown in Table 2, the mean age of the sample was 57.3 years (range of 29–79). Mean months since breast cancer diagnosis was 11.6 (range of 1–18 months). The sample was 74% white, 18% black, 4% Asian, 3% Hispanic, and 1% American Indian. Most (90%) had at least some college education. In terms of breast cancer treatment, 73% had lumpectomy (breast conserving surgery), 31% had mastectomy, 68% had radiation therapy, 43% had chemotherapy, 23% had surgery on the unaffected breast (contralateral mastectomy), and 77% had lymph nodes removed. There were five individuals who reported neither surgery option and 18 who reported having both types of surgery (likely because these patients had a mastectomy following a lumpectomy with unclear margins) which explains why the numbers for breast conserving surgery and mastectomy do not add up to 100%.

**Table 2:** **Sample description**

	**Mean/N**	**Range/%**
Age (mean (range))	57.3	(29–79)
Months since diagnosis (mean (range))	11.6	(1–18)
**Race/Ethnicity (%)**		
White	234	74.3
Black	57	18.1
Asian	13	4.1
Hispanic	8	2.5
American Indian	3	1.0
**Education (%)**		
HS or less	32	10.0
Some college	100	31.4
College or greater	187	58.6
**Treatment received (%)**		
Radiation therapy	218	68.3
Chemotherapy	138	43.1
Lumpectomy	233	72.8
Mastectomy	99	30.9

For overall BC treatment decisions, as well as for surgery, chemotherapy, and radiation decisions separately, the principal components analysis indicated a single factor solution, i.e., only one Eigen value greater than 1.0, and in each case the Eigen value for the single factor exceeded 2.4. As shown in Table 3, factor loadings of the six items were generally consistent across the overall and treatment-specific scales. The only item that did not meet the criteria for inclusion, based on factor loading >0.45, was “Right for You” for the chemotherapy and radiation scales.

**Table 3:** **Factor loadings for subjective decision quality items**

	**Factor loadings**			
**Item**	**Overall (n = 286)**	**Surgery (n = 286)**	**Chemotherapy (n = 195)**	**Radiation (n = 247)**
“Right for you”	0.49	0.50	0.25	0.25
Information	0.63	0.76	0.79	0.70
Time	0.77	0.81	0.81	0.65
Involvement	0.57	0.77	0.83	0.72
Regret	0.67	0.68	0.64	0.70
Satisfaction	0.59	0.72	0.73	0.74

Internal consistency (i.e., alpha) was 0.77, 0.85, 0.82, and 0.78 for the overall, surgery, chemotherapy, and radiation decision quality scales, respectively. The removal of “right for you’ slightly increased alpha for the chemotherapy and radiation scales. See Table 4.

**Table 4:** **Internal consistency (Alpha) for four subjective decision quality scales**

	**Overall (n = 286)**	**Surgery (n = 286)**	**Chemotherapy (n = 195)**	**Radiation (n = 247)**
Alpha with all items	0.77	0.85	0.82	0.78
Alpha if item removed				
“Right for you”	0.76	0.85	0.86	0.81
Information	0.75	0.82	0.77	0.74
Time	0.71	0.81	0.76	0.75
Involvement	0.75	0.82	0.76	0.73
Regret	0.73	0.82	0.78	0.74
Satisfaction	0.74	0.81	0.77	0.72

The three treatment-specific decision scale scores, computed from the means of the six items, formed a single factor with an eigenvalue of 1.75. The factor loadings for the three treatment-specific subscales on this single factor were .75, .76 and .79, for surgery, chemotherapy, and radiation, respectively. The correlations of the three subscale scores of decision quality with the overall decision quality score were .75, .70, and .74, respectively, for surgery, chemotherapy, and radiation. Data not shown.

Across the six items between 70% and 89% of respondents answered with the most positive category, i.e., completely right for me, no regret, or totally satisfied for the right for me, regret, and satisfaction items respectively, and “Just Right” for the information, involvement, and time items. Data not shown. As shown in Table 5, overall means for the four scales were, 6.45, 6.56, 6.33, and 6.55, respectively, for overall treatment, surgery, chemotherapy, and radiation. Mean scores for the surgery scale were significantly higher for those ages 58 and higher compared to those less than 58 years. Race/Ethnicity was associated with scores of the surgery and chemotherapy scales. Specifically, African American women had significantly (p < .05) lower scores than whites for surgery decision quality and both African American and Hispanic women had significantly (p > .05) lower scores than whites for chemotherapy decision quality. Months since diagnosis, procedures received (with one exception of surgery scores by chemotherapy), and education were not related to scale scores.

**Table 5:** **Scale means by patient demographic and health factors**

	**Overall**		**Surgery**		**Chemotherapy**		**Radiation**	
	**N**	**Mean**	**N**	**Mean**	**N**	**Mean**	**N**	**Mean**
**Scale mean**	298	6.45	295	6.56	207	6.33	255	6.55
**Age**								
< 58	148	6.40	147	6.41^1^	114	6.25	122	6.52
> =58	150	6.49	148	6.70^1^	193	6.44	133	6.58
**Months since diagnosis**								
< 12	131	6.52	130	6.61	83	6.35	114	6.65
> =12	167	6.39	165	6.51	124	6.32	141	6.48
**Race/Ethnicity**								
White	222	6.50	220	6.62^1^	153	6.50^1,2^	186	6.62
Black	50	6.24	49	6.20^1^	33	5.76^1^	45	6.33
American Indian	3	6.89	3	6.94	3	6.61	3	6.61
Asian	12	6.61	12	6.71	9	6.31	11	6.60
Hispanic	7	6.33	7	6.52	5	5.33^2^	6	6.22
**Education**								
HS or less	27	6.59	27	6.70	16	6.18	25	6.55
Some college	90	6.46	92	6.52	66	6.32	85	6.44
College or greater	181	6.42	176	6.55	125	6.36	145	6.62
**Treatment received**								
Radiation therapy								
Yes	206	6.46	206	6.56	143	6.36	210	6.59
No	88	6.43	85	6.54	61	6.28	42	6.36
Chemotherapy								
Yes	128	6.37	133	6.38^1^	132	6.26	112	6.49
No	166	6.50	159	6.70^1^	73	6.46	140	6.61
Lumpectomy								
Yes	219	6.48	215	6.61	145	6.37	214	6.57
No	75	6.38	76	6.42	59	6.25	39	6.52
Mastectomy								
Yes	92	6.35	93	6.36	73	6.24	54	6.39
No	201	6.48	197	6.64	131	6.37	197	6.59

Values with common superscript differ significantly, t test p value < .05.

## Discussion

The primary finding from these analyses is that our measure of subjective appraisal of breast cancer treatment decisions appears to comprise at least five related dimensions. These five dimensions, regret and satisfaction as well as adequacy of information, time, and involvement appear to merit inclusion on the final scale for the overall treatment as well as treatment-specific scales (i.e., surgery, radiation, and chemotherapy). The remaining element “Right for You”, was a candidate for removal, based on factor loading and/or internal consistency results for decisions regarding chemotherapy and radiation. For overall treatment and surgery specific decisions, however, it appears to merit inclusion. Although for parsimony we recommend the five –item version, for investigators assessing decision quality for chemotherapy and radiation for breast cancer, inclusion of the “right for you” item may be justified.

A second finding is that scores from the three treatment-specific decisions scales correlated with overall decision quality in the range of .70 to .75, with surgery exhibiting the highest correlation with the overall score. This raises the question as to whether decision quality should be assessed for each treatment separately or only for the patient’s overall treatment experience. On one hand, given these correlations, each treatment appears to capture a somewhat independent picture of decision quality. However if questionnaire space is limited and/or respondent burden is a particular concern, measuring only the overall treatment experience may be appropriate.

The overall means of the four scales were high; 6.3 or higher (out of 7) and across the six items between 70% and 89% of respondents answered with the most positive category, i.e., “completely right for me”, “no regret”, and “totally satisfied” for the right for me, regret, and satisfaction items respectively, and “just right” for the information, involvement, and time items. This positively skewed distribution (i.e., ceiling effect) is consistent with numerous prior studies both for decision ratings for breast cancer [35-40] and other cancers [37,41,42]. These high scores may be, at least in part, inflated by patient response bias, e.g., patients’ desire/need to feel good about their decisions to minimize dissonance. Determining how much patient bias may influence decision quality scores is an important area of research that cannot be answered from our data. At one site surveys were administered in person at the clinic and at the other site via mail. We therefore examined scale means by study site but found no significant differences for any of the four scales. Both sites provide highly specialized breast cancer treatment which could have biased scores upward. Whether surveying patients from less specialized treatment centers would have resulted in lower scores cannot be answered given our study sample. Because this is a measure of subjective decision quality, the values we observed may be considered inherently valid. Nonetheless, understanding how, if at all, positive reporting bias and/or social desirability may contribute to these scores remains an important area of future research. Additionally, strategies to reduce positive respondent bias, perhaps by altering our item stems or response categories, merit attention.

The association of treatment-specific scores with overall decision quality scores was slightly stronger for surgery than for chemotherapy and radiation. On one hand, this may indicate that surgery experiences contribute more to patients’ overall treatment appraisal than for chemotherapy and radiation. On the other hand, because surgery items were placed before chemotherapy and radiation on the survey, it may be that ordering effects contributed to these correlations. It should be noted that to minimize order effects, we placed the overall item before surgery, chemotherapy, and radiation for information, involvement, and satisfaction sections and afterwards for “right for you”, time, and regret. When the treatment-specific items preceded the overall items, the mean of those items were correlated .67 with the mean of the overall items, whereas when the treatment-specific items followed the overall items the means were correlated .75 with overall. Thus, a small ordering effect for when the overall experience and treatment-specific experiences were rated was evident in our sample. However, we did not vary the order of the surgery, chemotherapy, and radiation-specific items within each section, so the impact of ordering effects on these dimensions cannot be determined. Studies using random ordering of all items may be useful to determine the full impact of ordering on subject responses.

The initial psychometric properties of the scale is encouraging and, like other subjective rating scales, these items may be considered inherently valid as they tap patient opinion or feeling. More expansive exploration regarding the validity of our decision quality measure, however, is still warranted. This could include examining how our measure may be associated with outcomes such as quality of life, cancer-related anxiety, and perseveration and personality attributes such as social desirability and optimism. It is somewhat noteworthy that scale scores did not vary by time since diagnosis when we split the sample at before/after 12 months. There is limited work evaluating patients’ perceptions of breast cancer treatment decisions for periods of time greater than one year following diagnosis. Our survey did not include objective measures of decision quality, i.e., treatment knowledge, values concordance, or decisional concordance. Examining the relationship between objective and subjective measures of decisional quality is encouraged.

We identified five to six dimensions of subjective decision quality that merited inclusion in our final scale; however, other domains not measured here may also be important components of decision quality. For example, subjective difficulty, perseveration, conflict, decision and/or social support, and anxiety related to cancer treatment decisions may be important elements of quality decisions. They may load on the single dimension identified here or they may yield additional factors. We limited our scale to these six dimensions in part to keep our scales brief, although this tradeoff may have come at the cost of a more comprehensive and perhaps multi-dimensional measure of the construct.

The study sample had relatively high levels of education, with 90% having at least some college. Although there were no differences in scale scores by level of education, the sample size for the lower education segment was small. How the scale may perform among a lower educated and perhaps lower literacy population should be examined. African American and Hispanic women had lower decision quality scores for surgery and chemotherapy than other groups. These differences remained after adjustment for age and education. Elucidating why these (and perhaps other) groups report low decision quality merits exploration and may indicate potential opportunities for intervention or policy change.

Finally, we did not conduct cognitive interviews with patients for this psychometric pilot. Although we have used these items and the corresponding response scales in prior studies and obtained respondent feedback, it would have been useful to conduct further pretesting including cognitive interviewing to better elucidate how the items are perceived. This is a priority for our future research.

## Conclusions

Appraisal of BC treatment decisions is comprised of five related elements; regret and satisfaction as well as perceived adequacy of information, time, and involvement. Assessment of the quality of each treatment decision (e.g., surgery, radiation, chemotherapy) separately is recommended when feasible. Other domains not measured in our study (e.g., subjective difficulty, conflict, or anxiety) may also be important elements of a quality decision. The initial promising psychometric properties of this brief measure of subjective quality decision suggests it may be a useful tool for understanding patients’ perceptions of their breast cancer treatment decisions and could be used in research and practice.

Future psychometric testing is encouraged, including establishing norms for the scale as well as its convergent and divergent validity by examining how the measure is associated with other outcomes such as quality of life, psychological coping, decisional conflict, objective decision quality, and perhaps even survival. Although we tested the measure in the context of breast cancer treatment, the scale may be applicable to assessing subjective decision quality for other preference sensitive medical decisions.
